# Effects of Gestational Diabetes Mellitus on Cholesterol Metabolism in Women with High-Risk Pregnancies: Possible Implications for Neonatal Outcome

**DOI:** 10.3390/metabo12100959

**Published:** 2022-10-11

**Authors:** Aleksandra Zeljković, Daniela Ardalić, Jelena Vekić, Tamara Antonić, Sandra Vladimirov, Manfredi Rizzo, Tamara Gojković, Jasmina Ivanišević, Marija Mihajlović, Sanja Vujčić, Petar Cabunac, Vesna Spasojević-Kalimanovska, Željko Miković, Aleksandra Stefanović

**Affiliations:** 1 Department of Medical Biochemistry, Faculty of Pharmacy, University of Belgrade, 11000 Belgrade, Serbia; 2 Gynecology and Obstetrics Clinic Narodni Front, 11000 Belgrade, Serbia; 3 Department of Health Promotion, Mother and Child Care, Internal Medicine and Medical Specialties, University of Palermo, 90100 Palermo, Italy; 4 Department of Gynecology and Obstetrics, Faculty of Medicine, University of Belgrade, 11000 Belgrade, Serbia

**Keywords:** high-risk pregnancy, gestational diabetes, cholesterol synthesis and absorption, LDL and HDL particles, neonatal outcome

## Abstract

Metabolic disorders in pregnancy, particularly gestational diabetes mellitus (GDM), are associated with an increased risk for adverse pregnancy outcome and long-term cardiometabolic health of mother and child. This study analyzed changes of serum cholesterol synthesis and absorption markers during the course of high-risk pregnancies, with respect to the development of GDM. Possible associations of maternal lipid biomarkers with neonatal characteristics were also investigated. The study included 63 women with high risk for development of pregnancy complications. Size and proportions of small low-density (LDL) and high-density lipoprotein (HDL) particles were assessed across trimesters (T1–T3), as well as concentrations of cholesterol synthesis (lathosterol, desmosterol) and absorption markers (campesterol, β-sitosterol). During the study, 15 women developed GDM, while 48 had no complications (non-GDM). As compared to the non-GDM group, women with GDM had significantly higher triglycerides in each trimester, while having a lower HDL-C level in T3. In addition, they had significantly lower levels of β-sitosterol in T3 (*p* < 0.05). Cholesterol synthesis markers increased across trimesters in both groups. A decrease in serum β-sitosterol levels during the course of pregnancies affected by GDM was observed. The prevalence of small-sized HDL decreased in non-GDM, while in the GDM group remained unchanged across trimesters. Newborn’s size in the non-GDM group was significantly higher (*p* < 0.01) and inversely associated with proportions of both small, dense LDL and HDL particles (*p* < 0.05) in maternal plasma in T1. In conclusion, high-risk pregnancies affected by GDM are characterized by altered cholesterol absorption and HDL maturation. Advanced lipid testing may indicate disturbed lipid homeostasis in GDM.

## 1. Introduction

Pregnancy is a unique physiological state that is normally associated with alterations of glucose metabolism. Accordingly, the main feature of early pregnancy is hyperinsulinemia, while progressive insulin resistance of peripheral tissues is the common characteristic of late pregnancy [1]. Such changes of maternal glucose homeostasis are adaptive and have a critical importance for normal growth and development of the fetus. Development of gestational diabetes mellitus (GDM), on the other hand, indicates a severe disorder of glucose metabolism, and it is therefore associated with an increased risk of complications for both mother and newborn [2]. Among others, women with GDM are at higher risk of hypertensive disorders of pregnancy, such as preeclampsia or eclampsia [3].

Throughout gestation, maternal adaptive response also results in apparent changes of lipid metabolism. Alterations of lipid profile during the course of normal pregnancy are transient and include increase in triglycerides (TG), low-density lipoprotein cholesterol (LDL-C) levels, and high-density lipoprotein cholesterol (HDL-C) levels [4,5,6]. Since glucose metabolism disorders are intimately associated with dyslipidemia, impaired glucose tolerance in GDM further affects lipoprotein metabolism. Evidence suggests that the increase in pro-atherogenic lipid parameters, predominantly TG levels, is more prominent in early pregnancy of women who developed GDM, as compared to controls, while the data for LDL-C concentration are less conclusive [7,8]. Furthermore, an obvious reduction of HDL-C level was usually reported during the third trimester of pregnancies associated with GDM development [7]. In addition, GDM is characterized by changes of apolipoproteins’ levels. Recently, it has been demonstrated that serum and placental levels of apolipoprotein E (apoE) were decreased in pregnant women with GDM, and that this apolipoprotein’s concentrations negatively correlated with oxidative stress parameters in GDM [9]. Similarly, apolipoproteins AII, CI, and CIII were reportedly decreased in women with GDM [10]. Importantly, it has been found that postpartum levels of apoCIII and indices apoCIII/apoAI, apoCIII/apoAII, apoCIII/apoCII, and apoCIII/apoE are positively associated with the risk for development of type 2 diabetes in women with previous GDM [11]. It should also be mentioned that previous results have shown that administration of apolipoprotein AI can ameliorate insulin resistance in pregnant rats [12]. However, similar findings were not confirmed in the case of human pregnancies [13].

Plasma cholesterol pool is firmly controlled at multiple points, involving cholesterol absorption, synthesis, and excretion, and each of these processes can be dysregulated in patients with glucose metabolism disorders. Valuable information on cholesterol homeostasis can be provided by the assessment of non-cholesterol sterols in plasma [14]. Non-cholesterol sterols (NCSs) include different steroid metabolites, such as cholesterol precursors and plant sterols, which serve as novel biomarkers of cholesterol biosynthesis and intestinal absorption efficiency, respectively [15]. In our previous study, we demonstrated a disturbed cholesterol homeostasis among women with preeclampsia, in comparison to those with high-risk pregnancies [16]. Yet, only one study so far investigated non-cholesterol sterols in women with GDM and showed increased levels of cholesterol synthesis biomarkers, as compared to non-diabetic pregnant women [17]. No data, however, exist regarding the changes of cholesterol homeostasis indices in women with high-risk pregnancies with respect to the development of GDM. Furthermore, it is still insufficiently explored as to whether alterations of cholesterol synthesis and absorption processes following diagnosis of GDM have a potential impact on neonatal outcome. Better understanding of these processes could potentially improve clinical evaluation of women with pregnancy complications. Namely, cholesterol synthesis and absorption markers might be useful as novel biomarkers of increased risk for undesirable pregnancy outcome. Moreover, maintaining an adequate cholesterol homeostasis during pregnancy might be defined as a therapeutic target as well.

Although maternal glucose tolerance is usually normalized after pregnancy, women with GDM have a substantially increased risk of developing type 2 diabetes mellitus later in life [18]. In addition, recent clinical practice guidelines acknowledge that high-risk pregnancies significantly contribute to future cardiovascular disease risk [19]. Finally, according to the concept of Developmental Origins of Health and Disease (DOHaD), adverse intrauterine condition, due to either maternal hyperglycemia or dyslipidemia, might have an adverse impact on the future cardiometabolic health of the offspring [20]. These data indicate that prevention, timely recognition, and management of the disorders of glucose and cholesterol homeostasis during pregnancy are of critical importance for future cardiometabolic health of both mother and child.

The aim of this study was to explore characteristics of cholesterol metabolism in high-risk pregnancies affected by GDM. Moreover, our intention was to explore whether changes in maternal cholesterol homeostasis during pregnancy are associated with any alterations in the newborn’s weight, height, head circumference, and APGAR score.

## 2. Materials and Methods

### 2.1. Subjects

This study enrolled 63 pregnant women with one or more risk factors for development of pregnancy complications and adverse outcomes, who were referred by their primary gynecology health-care professional to the Gynecology and Obstetrics Clinic Narodni Front, Belgrade, Serbia. Initially, we recruited 92 pregnant women with a high risk for pregnancy complication development, according to the recommendations given by the American College of Obstetricians and Gynecologists (ACOG) and the National Institute for Health and Care Excellence (NICE, London, UK) [21,22]. The recruited subjects had one high-risk factor (comprising chronic hypertension, chronic kidney disease, hypertension in previous pregnancy, high uterine artery pulsatility index, diabetes, autoimmune diseases, antiphospholipid syndrome, or thrombophilia) or two moderate-risk factors (maternal age of 40 or older, pregnancy interval > 10 years, body mass index (BMI) > 30 kg/m^2^ before pregnancy, and family history of preeclampsia) for pregnancy complication development. Exclusion criteria were multifetal pregnancy, miscarriage, infectious disease or exacerbation of the existing autoimmune disease at any point during pregnancy, and malignant disease before pregnancy. A total of 48 out of 92 participants finished their pregnancies without any complication and they formed the control (non-GDM) group. A total of 15 women developed GDM and the remaining 29 women had other complications, but were non-GDM. In the GDM group, six cases developed preeclampsia in parallel with GDM, three cases had GDM and intrauterine growth restriction, two cases had hypertension alongside GDM, and four cases had GDM as the only complication.

Maternal characteristics (gestational age, age, height, weight, smoking status) and general obstetrics and medical history were assessed through the interpersonal interview and medical examination. Blood pressure was measured, and the mean arterial pressure was calculated by standardized protocol [23]. Color Doppler ultrasonography was used for the mean pulsatility index calculation [24]. All participants were encouraged to use vitamin and antioxidative supplementation recommended for pregnant women. No subjects were treated with lipid-lowering therapy.

The participants were informed in detail about the study aims and the study protocol, and all provided written consent for participation. This study was approved by the Ethics Committee of the Faculty of Medicine, University of Belgrade; the Ethics Committee of the Faculty of Pharmacy, University of Belgrade; and the Ethics Committee of the Gynecology and Obstetrics Clinic Narodni Front (Belgrade, Serbia) and conducted in accordance with the Declaration of Helsinki.

### 2.2. Sampling

This study was designed as a longitudinal study. Blood samples were taken toward the end of each trimester (first trimester (T1; 11–13 weeks of gestation), second trimester (T2; 20–23 weeks of gestation), third trimester (T3; 28–32 weeks of gestation)) after night-time fasting. Samples were centrifuged at 1500× *g* for 10 min to obtain serum and plasma and were aliquoted and stored at −80 °C until analysis.

### 2.3. Biochemical Analyses

Serum glucose, TG, total cholesterol (TC) and HDL-C, apolipoprotein AI (apoAI), and apolipoprotein B (apoB) were measured by commercial kits (Brea, CA, USA, Beckman Coulter) on an AU480 Chemistry Analyzer (Beckman Coulter). LDL-C concentration was calculated according to the Friedewald equation.

### 2.4. Determination of Advanced Lipid Profile Parameters

Quantification of NCSs was performed by liquid chromatography–tandem mass spectrometry (LC–MS/MS), as previously described in detail [25]. Briefly, serum samples were treated with KOH ethanolic solution and then underwent liquid extraction with n-hexane/water (4:1, *v*/*v*). After drying under nitrogen, the extract was reconstituted in methanol and further processed by LC–MS/MS. NCS separation was achieved by using a Poroshell 120 EC column (Agilent Technologies (USA)) and acetonitrile/methanol/water with 0.1% formic acid (80/18/2, *v*/*v*/*v*) as a mobile phase. Quantification of NCSs was performed by Mass Hunter software on an Agilent 1290/6420 LC–APCI–MS/MS (Agilent Technologies (Santa Clara, CA, USA)). Lathosterol and desmosterol were quantified as cholesterol synthesis markers, whilst campesterol and β-sitosterol as cholesterol absorption markers. The sum of concentrations of both cholesterol synthesis markers was designated as a measure of cholesterol synthesis, and the sum of campesterol and β-sitosterol concentrations was defined as a measure of cholesterol absorption. Cholesterol synthesis/absorption ratio was calculated by dividing cholesterol synthesis and absorption levels.

Determination of LDL and HDL particle size and relative abundance of smaller LDL and HDL subfractions was performed by polyacrylamide gradient gel electrophoresis, according to a previously established method [26]. In short, 3–31% polyacrylamide gels were used for electrophoretic separation of LDL (LDL I-LDL IV) and HDL (HDL2b-HDL3a) subclasses by employing a Hoefer SE 600 Ruby electrophoresis unit (Amersham Pharmacia Biotech, Vienna, Austria). Relative proportions of specific LDL or HDL subclasses were determined according to corresponding fractions of densitometric scans of electropherograms, which were obtained by using an Image Scanner Image Scanner (Amersham Pharmacia Biotech, Vienna, Austria) and Image Quant software (version 5.2; 1999; Molecular Dynamics Inc., Sunnyvale, CA, USA). LDL and HDL particle sizes were assessed by the determination of diameters of the highest absorbance peaks in LDL and HDL regions of the densitometric scan. Relative proportion of sdLDL was calculated as a sum of percentages attributed to all LDL subclasses with diameters ≤ 25.5 nm. Accordingly, relative proportion of small-sized HDL was assessed as a sum of percentages belonging to HDL subclasses with diameters ≤ 8.8 nm.

### 2.5. Statistical Analysis

Normally distributed continuous (according to the Kolmogorov–Smirnov test) data are presented as mean ± standard deviations, while asymmetrically distributed variables are presented as median (interquartile range). Categorical data are presented as absolute frequencies. Group differences were tested by Student’s *t*-test or the Mann–Whitney U-test for continuous variables, depending on the type of data distribution. Group differences for categorical data were tested by the chi-squared test. Changes in the examined variables across trimesters of pregnancy were tested by the Paired *t*-test for normally distributed variables, or the Wilcoxon signed rank test for variables with skewed distribution. Correlation analysis was assessed by using Spearman’s correlation coefficient. All statistical tests were performed by SPSS version 22.0 for Windows statistical package (SPSS, Chicago, IL, USA). Significant differences were considered if *p* < 0.05.

## 3. Results

Baseline characteristics of the study participants are presented in Table 1. There were no differences in subjects’ age, pre-gestational weight, pre-gestational BMI, or smoking status between the groups with and without development of GDM. The prevalence of first-time pregnancies was similar in both groups.

Next, we compared general clinical and biochemical characteristics of GDM and non-GDM groups in each trimester of pregnancy (Table 2). We found no differences in BMI; weight increase; glucose level; and TC, LDL-C, apoAI, and apoB concentrations among the groups in any of the gestational trimesters. Blood pressure values were comparable among the groups, except for higher DBP in the GDM group in T1. In contrast, TG concentrations were consistently higher in the GDM group in every trimester. In addition, HDL-C level was significantly lower in the GDM group, but only in T3.

To further explore lipid profile changes in GDM, we analyzed differences in NCS levels between the two examined groups (Table 3). There were no differences in any of the determined NCSs, except for a significantly lower level of β-sitosterol in women with GDM in T3. Accordingly, total amount of cholesterol absorption markers in T3 was lower in the GDM group, although with borderline statistical significance.

Additionally, we examined longitudinal alterations in cholesterol synthesis and absorption markers across trimesters in pregnant women who did and did not develop GDM (Table 4). Levels of NCSs in T1 were used as a reference group. The obtained results demonstrated that lathosterol levels were higher in T2 and T3 when compared to T1 in both GDM and non-GDM groups. Similarly, levels of desmosterols were raised across trimesters in both analyzed groups. Accordingly, summary levels of cholesterol synthesis increased across trimesters in both GDM and non-GDM subjects. Analysis of cholesterol absorption markers revealed that campesterol concentrations in T2 and T3 were similar to that in T1 in both groups. Although differences in β-sitosterol levels did not reach statistical significance, we noticed a trend of decreasing concentrations of this absorption marker in T2 and T3 in the GDM group, but not in the non-GDM group. Lastly, the cholesterol synthesis/absorption ratio was higher in T2 and T3 when compared to T1 in both groups.

Further, we analyzed possible changes in LDL and HDL particle sizes and prevalence of small HDL and HDL particles across trimesters (Table 5). When compared to T1, LDL particle size was decreased in T2 and T3 in both pregnant women with and without GDM, while HDL particle diameters were comparable. We found no differences in relative proportion of sdLDL particles, but small-sized HDL particles were less prevalent in the non-GDM group in T2 when compared to T1.

Directions of the most prominent changes in advanced lipid profile across trimesters of pregnancies with and without GDM development are summarized in Table 6. Cholesterol synthesis and synthesis/absorption ratio increased, while LDL particle size decreased in both groups as pregnancy advanced. HDL particle size and prevalence of sdLDL remained stable in both groups across trimesters. We found no variations in cholesterol absorption during pregnancy without GDM development, while the proportion of small-sized HDL particles decreased in this group. In contrast, we noticed a trend of lowering of cholesterol absorption and abounding of smaller HDL particles in the GDM group, although statistical significance was not reached.

We present general newborn characteristics in both groups in Table 7. The only significant difference was found in newborns’ weight, which was significantly lower in the GDM group.

There were no differences in birth weight between neonates born to mothers who smoked before pregnancy and those born to mothers who never smoked (*p* = 0.885). Similar results were obtained when we compared newborns’ weight with respect to maternal smoking status in the GDM and non-GDM groups separately (*p* = 0.711, *p* = 0.646, respectively).

Correlation analysis did not show any significant correlation of cholesterol synthesis markers with cholesterol absorption markers, either in the GDM or in the non-GDM group (data not shown). We found a statistically significant negative correlation between newborn weight and relative proportion of small-sized HDL particles in T1 (ρ = −0.327; *p* = 0.040) and T2 (ρ = −0.354; *p* = 0.027) in the non-GDM group. No other lipid status marker correlated with newborn weight in both groups (data not shown). Newborn length was in significant negative correlation with LDL particle size in T1 (ρ = −0.338; *p* = 0.033) in the non-GDM group. We found significant negative correlation between newborn head circumference and relative proportion of small-sized HDL particles in T2 (ρ = −0.336; *p* = 0.037) in the non-GDM group. No significant correlation between lipid status markers and general newborns characteristics in both groups in T3 was found (data not shown).

## 4. Discussion

In this study, we sought for possible lipid status differences among high-risk pregnancies that were affected by the development of GDM or ended without any complications. According to our results, women with GDM had higher TG concentrations during the entire course of pregnancy and decreased HDL-C levels in the third trimester. Moreover, detailed lipid status analysis revealed differences in patterns of cholesterol synthesis and absorption between the two groups.

GDM is one of the leading pregnancy complications with possible adverse effects on pregnancy outcome, but also on long-term cardiometabolic health of both mother and child [27,28,29]. It is well known that metabolic adaptation to pregnancy is associated with the development of insulin resistance to ensure an adequate fetal supply with glucose. Hypertriglyceridemia is a common consequence of insulin resistance, but this relationship is bi-directional, since the evidence accumulated suggesting that dyslipidemia can contribute to insulin resistance development as well [30]. A recent systematic review and meta-analysis [31] revealed high TG levels as hallmarks of GDM-associated dyslipidemia, which is in accordance with our results (Table 2). The same research demonstrated higher levels of TC and LDL-C, while lower HDL-C concentrations in women with GDM. In our study, significantly lower HDL-C levels were found only in the third trimester. However, it should be noted that we analyzed differences in lipid status of women with GDM when compared to women with high-risk pregnancies that ended without complications, but not to completely physiologically healthy pregnancies. This could be a reason for discrepancy with the results of the above-mentioned meta-analysis. It is also noteworthy that not all studies demonstrated the same pattern of changes in serum lipids in GDM, as recently reviewed [31]. Thus, this topic should be further addressed to clarify the issue of routine lipid status changes in GDM-affected pregnancies.

In contrast to frequently analyzed routine serum lipid parameters in GDM, far less attention is dedicated to the investigation of cholesterol synthesis and absorption processes in this medical condition. The results of our study have shown that pregnancies with and without GDM are both characterized by a gradual increase in cholesterol synthesis across trimesters (Table 4). A rise in maternal cholesterol is typically seen in pregnancy and is aimed at fulfilling the requirements of the fetus [32]. Previously, Miettinen et al. [17] demonstrated elevated cholesterol synthesis in obese pregnant women with GDM, when compared with their non-GDM counterparts. However, these conclusions are mainly derived from elevated squalene levels, while the authors did not find differences in other cholesterol synthesis markers, similarly to what we demonstrated herein (Table 3). The lack of significant differences in our study might be explained by the fact that all analyzed cases belonged to high-risk pregnancies, indicating already disturbed lipid homeostasis. Keeping in mind limited data on cholesterol synthesis markers in GDM-affected pregnancies so far, it remains to be established as to whether the pattern of cholesterol synthesis is significantly changed in GDM or not. Moreover, variations of cholesterol metabolism in high-risk pregnancies should not be neglected, even if they are not affected by any complications, since possible adverse effects on long-term maternal and neonatal health are possible, regardless of the immediate pregnancy outcome.

The obtained results regarding cholesterol absorption were somewhat unexpected. Namely, in physiological conditions, cholesterol synthesis and absorption processes are balanced, so increased endogenous synthesis is followed by decreased intestinal absorption of exogenous cholesterol [15]. However, in the present study, we found no changes in cholesterol absorption markers across trimesters in the high-risk group that was not affected by GDM development. On the other hand, we noticed a trend of lower cholesterol absorption in the GDM group, although statistical significance was not reached (Table 4). The observed differences were mostly related to the levels of β-sitosterol, and we found a statistically significant decrease in this cholesterol absorption marker in the third trimester in GDM subjects when compared to the non-GDM group (Table 3). Thus, even though cholesterol synthesis/absorption ratio was rising as pregnancies advanced in both groups, the pattern of simultaneous changes in these processes was somewhat different among GDM and non-GDM subjects, especially when it comes to cholesterol absorption. Previous analyses have pointed towards the absorption markers as indicators of maternal–fetal cholesterol transport as well [33,34], demonstrating lower levels of plant sterols in umbilical cord blood of neonates with intrauterine growth restriction [35]. Herein, we demonstrated lower levels of cholesterol absorption in mothers with GDM, which might suggest decreased fetal supply by cholesterol, even though endogenous cholesterol synthesis was elevated. Indeed, neonatal weight was significantly lower in the GDM group (Table 1), thus supporting the hypothesis that impaired maternal cholesterol metabolism could affect fetal growth. Moreover, it has been postulated that HDL particles play a crucial role in maternal–fetal cholesterol transport [36]. We found decreased prevalence of small-sized HDL particles in the non-GDM group across trimesters (Table 5), indicative for an enhanced process of HDL maturation in response to elevated cholesterol synthesis and absorption. On the other hand, the prevalence of small-sized HDL in the GDM group remained stable across trimesters. Hence, decreased cholesterol absorption and consequent inadequate HDL maturation can contribute to diminished fetal cholesterol supply in GDM. The observed negative correlation between the amount of small-sized HDL particles and newborn weight and newborn head circumference in the high-risk group unaffected by GDM confirmed such an assumption. A similar trend was observed in the GDM group, although without statistical significance. In our previous study, we demonstrated a negative correlation between newborn head circumference and the relative proportion of HDL 2a particles, i.e., HDL moieties that did not reach full maturation [5]. The currently obtained findings in high-risk pregnancies confirm and extend such assumptions. Of note, GDM is principally associated with neonatal macrosomia, and hence our results of reduced neonatal weight in this group might be considered as atypical. One possible reason for such findings could be heterogeneity of the GDM group, since other complications, including preeclampsia and hypertension, were also present in several cases. Yet, intrauterine growth restriction due placental dysfunction is also seen in GDM [37], so we cannot exclude GDM as a significant contributing factor to the smaller birth weight.

It is also important to mention that pregnancy-associated dyslipidemia can contribute to inadequate lipoprotein metabolism as well. Indeed, hypertriglyceridemia is associated with elevated presence of both sdLDL and small-sized HDL in various pathological conditions [38]. A rise in TG level was more prominent in the GDM group (Table 2), and this condition might enhance formation of sdLDL, but also accumulation of small-sized dysfunctional HDL.

The results presented herein emphasize the importance of pre-gestational and gestational maternal health for both immediate and long-term pregnancy outcomes. Since pharmacological therapeutic options for dyslipidemia in pregnancy are very limited, lifestyle modifications represent the first-line treatment. It is well known that a healthy diet rich in vegetables, micronutrients, fibers, and poly-unsaturated fatty acids, as well as moderate physical activity and a supportive psychological environment, can significantly reduce the risk for gestational complications and improve maternal and fetal outcomes [39]. The therapeutic approach in women with GDM is focused on ensuring healthy dietary habits to avoid hyperglycemia, as well as moderate (30 min daily) physical activity [40]. Such measures can improve lipid status as well. Of note, maintaining desirable lifestyle habits should not be limited to the period of gestation, since numerous evidence suggests that preconception health could be even more important for ensuring favorable pregnancy course and outcome [41]. Interestingly, we did not detect any significant differences in newborns’ weight with respect to maternal smoking habits, although a firm relationship between smoking before and during pregnancy and adverse pregnancy outcomes was established by many previous studies [42,43]. Such atypical results could arise due to a small sample size in our study. In addition, the fact that all study participants quit smoking when their pregnancy test was positive could also be the reason for lack of differences in neonates’ birth weight between the groups.

Recently, more attention has been paid to the impact of gut microbiota on overall metabolic changes, but data are inconsistent regarding the role of microbiome alterations in pregnancy [40]. However, this topic could be important, since it has been shown that gut microbiota can affect the plasma cholesterol level by several mechanisms [44,45]. Previous studies suggested the influence of probiotics on the reduction of cholesterol absorption [45]. Thus, considering our current findings, the possibility of using probiotics in the treatment of pregnancy-associated dyslipidemia should be evaluated by future studies. In line with this, cholesterol synthesis and absorption markers could potentially be useful as indicators of adverse changes in cholesterol homeostasis during the course of pregnancy with complications. Moreover, these parameters might well provide valuable information on the efficiency of the applied therapy.

Several limitations should be mentioned. First, small sample size limits statistical power of analyses, and it is possible that several observed trends would reach a statistical significance in a larger sample. However, our preliminary findings might direct future large-scale investigations. These initial results suggest that development of GDM during the course of high-risk pregnancy is associated with noticeable changes in cholesterol metabolism, which might impact fetal growth. Further investigations of differences in lipid homeostasis between healthy and GDM-affected pregnancies are needed to firmly establish which characteristics of cholesterol metabolism are typical for GDM. Finally, the GDM group was not homogenous, since several of cases were affected in parallel by other pregnancy complications. Thus, additional cautiousness is needed for the interpretation of the obtained results.

In conclusion, the results presented herein demonstrated variations in cholesterol metabolism, especially the cholesterol absorption process in pregnancies affected by GDM, alongside consequent changes in LDL and HDL size and distribution. The obtained findings suggest a possibility of using specific advanced lipid status parameters as indicators of disturbed cholesterol homeostasis in pregnancy with complications, but also as markers of inadequate fetal development. Future large-scale studies designed to further explore the raised hypotheses are warranted.

## Figures and Tables

**Table 1:** Baseline characteristics of study participants.

Parameter	GDM (*n* = 15)	Non-GDM (*n* = 48)	*p*
Age (years)	33 ± 4.05	31.73 ± 5.40	0.405
Pre-gestational weight (kg)	70.07 ± 18.24	67.18 ± 12.19	0.482
Pre-gestational BMI (kg/m^2^)	25.55 ± 5.68	23.98 ± 4.30	0.257
Smoking before pregnancy (yes/no)	4/11	15/33	0.736

Data were presented as means ± standard deviations and as absolute frequencies. Continuous variables were compared by Student’s t-test and categorical variables by chi-squared test.

**Table 2:** Differences in weight increase and serum lipid parameters among pregnant women with and without GDM across trimesters.

Parameter	T1			T2			T3		
	GDM	Non-GDM	*p*	GDM	Non-GDM	*p*	GDM	Non-GDM	*p*
Weight increase (%)	3.0 (0.5–4.7)	4.9 (2.0–6.2)	0.128	8.0 (5.0–10.0)	8.0 (6.0–10.0)	0.903	5.0 (3.0–6.0)	4.5 (3.0–6.0)	0.636
TC (mmol/L)	5.61 ± 1.13	5.24 ± 1.01	0.229	6.77 ± 1.48	6.77 ± 1.35	0.982	6.93 ± 1.33	7.45 ± 1.63	0.270
LDL-C (mmol/L)	2.88 ± 0.88	2.87 ± 0.83	0.945	3.58 ± 1.54	3.80 ± 1.13	0.533	3.72 ± 1.43	4.27 ± 1.30	0.168
HDL-C (mmol/L)	1.97 ± 0.64	1.80 ± 0.30	0.153	2.11 ± 0.39	2.10 ± 0.39	0.930	1.73 ± 0.33	2.08 ± 0.56	**0.025**
TG (mmol/L)	1.67 ± 0.83	1.26 ± 0.40	**0.012**	2.35 ± 0.88	1.91 ± 0.53	**0.021**	3.27 ± 0.93	2.43 ± 0.75	**0.001**
apoAI (g/L)	2.00 ± 0.34	1.93 ± 0.30	0.457	2.40 ± 0.43	2.29 ± 0.33	0.311	2.14 ± 0.33	2.29 ± 0.39	0.165
apoB (g/L)	1.09 ± 0.29	0.99 ± 0.21	0.168	1.28 ± 0.35	1.33 ± 0.31	0.609	1.43 ± 0.35	1.50 ± 0.41	0.538

Data are presented as means ± standard deviations and were compared by Student’s *t*-test. Bold represent to statistically significant *p* < 0.05.

**Table 3:** Differences in cholesterol synthesis and absorption markers among pregnant women with and without GDM across trimesters.

Parameter	T1			T2			T3		
	GDM	Non-GDM	*p*	GDM	Non-GDM	*p*	GDM	Non–GDM	*p*
Lathosterol (μmol/L)	11.25 (7.29–20.68)	11.52 (6.15–16.02)	0.402	16.15 (11.89–33.78)	15.87 (11.16–24.55)	0.789	21.31 (15.99–60.53)	24.90 (16.38–34.79)	0.617
Desmosterol (μmol/L)	1.74 (1.07–2.60)	1.60 (1.32–1.91)	0.558	1.83 (1.49–3.02)	1.92 (1.53–2.32)	0.980	2.23 (1.94–3.00)	2.49 (1.64–3.15)	0.872
Campesterol (μmol/L)	2.08 (0.80–4.70)	2.33 (1.98–3.01)	0.529	1.52 (1.05–4.04)	2.67 (1.87–3.21)	0.254	2.23 (1.36–3.28)	2.74 (1.90–3.85)	0.203
β-Sitosterol (μmol/L)	5.23 (4.14–10.03)	5.77 (4.30–6.60)	0.627	4.01 (3.45–10.55)	5.92 (4.53–6.89)	0.262	3.83 (3.68–6.25)	5.36 (4.05–7.36)	**0.045**
Cholesterol synthesis (μmol/L)	12.44 (9.04–23.29)	13.15 (7.56–17.96)	0.393	17.86 (14.47–36.81)	17.77 (13.17–27.24)	0.578	23.97 (18.03–34.48)	27.82 (18.53–42.23)	0.583
Cholesterol absorption (μmol/L)	7.16 (5.61–14.73)	8.10 (6.62–9.27)	0.384	5.51 (4.57–15.24)	8.58 (6.30–10.38)	0.519	6.05 (5.00–9.53)	8.30 (6.60–9.98)	0.066
Synthesis/absorption ratio	1.75 (0.98–3.29)	1.77 (0.82–2.41)	0.228	2.85 (1.15–5.57)	2.33 (1.73–2.99)	0.136	3.33 (2.21–5.23)	4.37 (2.09–4.91)	0.583

Data are presented as medians (interquartile ranges) and were compared by Mann–Whitney U-test. Bold represent to statistically significant *p* < 0.05.

**Table 4:** Pattern of changes in cholesterol synthesis and absorption across trimesters in pregnant women with and without GDM.

Parameter		T1	T2	T3
Lathosterol (μmol/L)	GDM	11.25 (7.29–20.68)	16.15 (11.89–33.78) **	21.31 (15.99–60.53) *
	Non-GDM	11.52 (6.15–16.02)	15.87 (11.16–24.55) ***	24.90 (16.38–34.79) ***
Desmosterol (μmol/L)	GDM	1.74 (1.07–2.60)	1.83 (1.49–3.02) *	2.23 (1.94–3.00)
	Non-GDM	1.60 (1.32–1.91)	1.92 (1.53–2.32) ***	2.49 (1.64–3.15) ***
Campesterol (μmol/L)	GDM	2.08 (0.80–4.70)	1.52 (1.05–4.04)	2.23 (1.36–3.28)
	Non-GDM	2.33 (1.98–3.01)	2.67 (1.87–3.21)	2.74 (1.90–3.85)
β-Sitosterol (μmol/L)	GDM	5.23 (4.14–10.03)	4.01 (3.45–10.55)	3.83 (3.68–6.25)
	Non- GDM	5.77 (4.30–6.60)	5.92 (4.53–6.89)	5.36 (4.05–7.36)
Cholesterol synthesis (μmol/L)	GDM	12.44 (9.04–23.29)	17.86 (14.47–36.81) **	23.97 (18.03–34.48) *
	Non-GDM	13.15 (7.56–17.96)	17.77 (13.17–27.24) ***	27.82 (18.53–42.23) ***
Cholesterol absorption (μmol/L)	GDM	7.16 (5.61–14.73)	5.51 (4.57–15.24)	6.05 (5.00–9.53)
	Non-GDM	8.10 (6.62–9.27)	8.58 (6.30–10.38)	8.30 (6.60–9.98)
Synthesis/absorption ratio	GDM	1.75 (0.98–3.29)	2.85 (1.15–5.57) *	3.33 (2.21–5.23) *
	Non-GDM	1.77 (0.82–2.41)	2.33 (1.73–2.99) ***	4.37 (2.09–4.91) ***

Data are presented as medians (interquartile ranges) and were compared by Wilcoxon signed rank test. * Significantly different from T1; *p* < 0.05. ** Significantly different from T1; *p* < 0.01. *** Significantly different from T1; *p* < 0.001.

**Table 5:** Pattern of changes in lipoprotein subclasses size and distribution across trimesters in pregnant women with and without GDM.

Parameter		T1	T2	T3
LDL particle size (nm)	GDM	26.56 ± 1.59	25.89 ± 1.73 **	24.57 ± 0.83 **
	Non-GDM	26.40 ± 0.91	25.82 ± 1.05 ***	25.38 ± 1.44 ***
SdLDL (%)	GDM	47.05 ± 11.13	46.67 ± 11.88	50.59 ± 9.51
	Non-GDM	48.52 ± 7.68	47.33 ± 10.05	46.55 ± 12.64
HDL particle size (nm)	GDM	10.29 ± 1.20	10.29 ± 0.98	10.43 ± 1.07
	Non-GDM	10.63 ± 0.92	10.60 ± 0.92	12.18 ± 10.30
Small-sized HDL (%) ^#^	GDM	25.70 (22.50–31.30)	27.60 (22.80–33.80)	28.85 (24.98–33.10)
	Non-GDM	28.00 (24.25–31.80)	26.00 (21.65–28.70) **	26.73 (22.11–30.70)

Data are presented as means ± standard deviations and were compared by paired t-test. ^#^ Data are presented as medians (interquartile ranges) and were compared by signed rank test. *p* < 0.05. ** Significantly different from T1; *p* < 0.01. *** Significantly different from T1; *p* < 0.001.

**Table 6:** Directions of changes of advanced lipid profile across trimesters.

Parameter		T1	T2	T3
Cholesterol synthesis (μmol/L)	GDM	●	↑	↑
	Non-GDM		↑	↑
Cholesterol absorption (μmol/L)	GDM	●	↓ ^#^	↓ ^#^
	Non-GDM		→	→
Synthesis/absorption ratio	GDM	●	↑	↑
	Non-GDM		↑	↑
LDL particle size (nm)	GDM	●	↓	↓
	Non-GDM		↓	↓
SdLDL (%)	GDM	●	→	→
	Non-GDM		→	→
HDL particle size (nm)	GDM	●	→	→
	Non-GDM		→	→
Small-sized HDL (%)	GDM	●	↑ ^#^	↑ ^#^
	Non-GDM		↓	↓ ^#^

●—reference group; ↑—increase; ↓—decrease; → —no changes; ^#^ Statistical significance was not reached for the observed trends. T1—reference group.

**Table 7:** Characteristics of the newborns of mothers with and without GDM.

Parameter	GDM (*n* = 15)	Non-GDM (*n* = 48)	*p*
Newborn’s weight (g)	3160.71 ± 483.25	3507.50 ± 326.51	**0.004**
Newborn’s length (cm)	50.3 ± 2.35	51.7 ± 3.71	0.204
Newborn’s head circumference (cm)	34.7 ± 1.03	35.1 ± 2.17	0.592
APGAR score (1 min) ^#^	9 (8–9)	9 (5–10)	0.139
APGAR score (5 min) ^#^	10 (8–10)	10 (6–10)	0.127

Data are presented as means ± standard deviations and were compared by Student’s *t*-test. ^#^ Data are presented as medians (interquartile ranges) and were compared by Mann–Whitney U-test. APGAR score: Appearance, Pulse, Grimace, Activity, and Respiration—scoring system for evaluation of a newborn’s condition. Bold represent to statistically significant *p* < 0.05.

## Data Availability

The data presented in this study are available on request from the corresponding author. The data are not publicly available due to privacy and ethics consideration.
